# Refixation behavior in naturalistic viewing: Methods, mechanisms, and neural correlates

**DOI:** 10.3758/s13414-023-02836-9

**Published:** 2024-01-02

**Authors:** Andrey R. Nikolaev, Radha Nila Meghanathan, Cees van Leeuwen

**Affiliations:** 1 Department of Psychology, Lund University, Box 213, 22100 Lund, Sweden; 2 Brain & Cognition Research Unit, KU Leuven–University of Leuven, Leuven, Belgium; 3 Department of Experimental Psychology, Otto-von-Guericke University, Magdeburg, Germany; 4 Center for Cognitive Science, Rheinland-Pfälzische Technische Universität Kaiserslautern-Landau, Kaiserslautern, Germany

**Keywords:** Eye movements, Gaze returns, Neural activity, Visual perception, Attention, Memory

## Abstract

When freely viewing a scene, the eyes often return to previously visited locations. By tracking eye movements and coregistering eye movements and EEG, such refixations are shown to have multiple roles: repairing insufficient encoding from precursor fixations, supporting ongoing viewing by resampling relevant locations prioritized by precursor fixations, and aiding the construction of memory representations. All these functions of refixation behavior are understood to be underpinned by three oculomotor and cognitive systems and their associated brain structures. First, immediate saccade planning prior to refixations involves attentional selection of candidate locations to revisit. This process is likely supported by the dorsal attentional network. Second, visual working memory, involved in maintaining task-related information, is likely supported by the visual cortex. Third, higher-order relevance of scene locations, which depends on general knowledge and understanding of scene meaning, is likely supported by the hippocampal memory system. Working together, these structures bring about viewing behavior that balances exploring previously unvisited areas of a scene with exploiting visited areas through refixations.

## Introduction

Natural viewing is a process of discovery. High-resolution visual sensing is limited to the fovea. The eyes are therefore constantly moving to progressively uncover the scene. This leads to the sequences of eye fixations known as scanpaths, studied by pioneering eye-tracking researchers (Buswell, 1935; Yarbus, 1967). Eye tracking reveals that the eyes often refixate—meaning they return to previously visited locations. This already caught the pioneers’ eyes. Alfred Yarbus (1967) described refixation behavior as follows:

If the eye movements are recorded for several minutes during perception of an object, the record obtained will clearly show that, when changing its points of fixation, the observer’s eye repeatedly returns to the same elements of the picture. Additional time spent on perception is not used to examine the secondary elements, but to reexamine the most important elements. The impression is created that the perception of a picture is usually composed of a series of “cycles,” each of which has much in common. (p. 193)

Our review will take another look at this behavior.

Refixations still have us wondering today. We might have imagined that the quickest and most parsimonious way to extract visual information from a scene would minimize refixations. Indeed, there are oculomotor mechanisms working against refixating. Inhibition of return (IOR) operates on successive eye movements to delay the gaze from returning to the location just visited (Klein, 2000). In addition, there is the well-known phenomenon of saccadic momentum; from one saccade to the next, the eyes tend to keep moving in the same direction (Smith & Henderson, 2009). Nevertheless, refixations constitute a significant proportion of natural viewing behavior: they amount to 10%–40 % of all fixations, depending on the visual task (M. Zhang et al., 2022). Their sheer number, as well as its task specificity, makes it unlikely that refixations are purely accidental.

Compared with locations to which the gaze does not return, refixated locations are more visually salient and task-relevant (Ballard et al., 1995; Wilming et al., 2013; M. Zhang et al., 2022). This as such does not make refixations special, as the eyes are habitually biased to visit salient and/or meaningful locations (Foulsham & Underwood, 2008; Koch & Ullman, 1985; Tatler et al., 2011). Refixations are special, we posit, because they are intimately related to the dynamics of memory during sequential eye movements. This tight relationship has even led researchers to use refixations as a probe for memory functions (Hollingworth & Bahle, 2020; Kragel et al., 2021). Accordingly, research on refixation behavior has relied heavily on theories of memory encoding, maintenance, and retrieval (Bays & Husain, 2012; Godwin et al., 2021; Hollingworth & Henderson, 2002; Maxcey-Richard & Hollingworth, 2013; Meghanathan et al., 2019; Shen et al., 2014; Wilming et al., 2013; Zelinsky et al., 2011; M. Zhang et al., 2022).

The principled understanding is that memory limitations lead to refixation behavior. When a representation decays over the course of visual exploration, refixating may be the way to rehearse the lost information (Zelinsky et al., 2011). Likewise when a memory representation of a previously visited location is overwritten by new information (Shen et al., 2014). But memory limitations could also indirectly affect our refixation behavior. Refixations can be included in a viewing strategy, a plan for inspecting the scene in order to perform a visual task efficiently within the constraints of memory. Such a strategy may involve a store/refixate trade-off. In certain interactive tasks involving object manipulation, locations are only refixated to obtain information immediately before it is needed (Droll & Hayhoe, 2007).

The store/refixate trade-off is encountered in natural viewing, where different locations compete for attention, but fixations on any of these locations can only be made serially, creating a bottleneck. This bottleneck is controlled by a competition between various locations according to their saliency and relevance, which could terminate a fixation before sufficient information has been sampled. Subsequent refixations then compensate for the deficit (Manohar & Husain, 2013; Peterson et al., 2001; H. Zhang et al., 2021).

A viewing strategy based on this principle could involve refixations in constructing the representation of an entire scene. As only a portion of the scene is sampled at each fixation, refixations facilitate information sampling by serving to accumulate and integrate information from different sampled items and their locations, to construct the scene representation (Kragel et al., 2021; Nikolaev, Bramão, et al., 2023; Pertzov et al., 2009; Tatler, Gilchrist, et al., 2005).

The viewing strategy thus is likely to involve an initial sequence of fixations on a scene to explore potentially important objects or locations to be revisited later. This makes it essential for eye-movement studies to consider the properties of the initial locations that are later revisited and the corresponding precursor fixations (Hooge et al., 2005; Nikolaev, Ehinger, et al., 2023; Wilming et al., 2013; M. Zhang et al., 2022).

When extensive inspection of a visual scene is called for, the viewing strategy of store/refixate trade-off may turn into one between exploring new regions and exploiting precursor locations (Gameiro et al., 2017). Exploration and exploitation have distinct scanpaths: the former is characterized by “ambient” viewing, with large saccades and short fixations, while the latter is characterized by “focal” viewing, characterized by small saccades and long fixations (Pannasch et al., 2008; Tatler & Vincent, 2008; Unema et al., 2005), and refixations (Wilming et al., 2013).

Although eye-tracking may offer a window into the complex brain processes underlying refixations, the high diversity of refixation functions makes it difficult to achieve even simple generalization across tasks (Zelinsky et al., 2011) using behavioral measures of eye movements alone. Nevertheless, M. Zhang et al. (2022) recently developed a universal computational model of refixations consisting of five major components: an image feature extractor, a saliency map, a target similarity map, constraints on saccade size, and an exponential memory decay. The decay implies that the model’s behavior rapidly approximates a memoryless system. Applied to the gaze patterns in eight human and animal gaze datasets, the model was able to stochastically predict spatiotemporal properties of various refixation behaviors, such as frequency, angle, preferred image locations, and the number of intervening fixations between the location to be revisited and the refixation. Despite this accomplishment, the authors acknowledge several limitations of their model related to memory functions, such as neglecting the target recognition system or the contribution of contextual information accumulated during viewing. These effects are systematic but insufficiently reflected in the stochastic patterns of eye movement. For this reason, our review will emphasize these memory functions in the dynamics of eye movement.

Our review is structured as follows. We begin with a definition of refixations and an overview of eye-tracking approaches used to study them, advocating an analysis of the recurrent dynamics of refixation behavior. Then we deal with the various roles refixations can play in a range of different tasks. We focus on what we will call the reparative, constructional, and strategic functions of refixations. The number and variety of factors affecting refixations suggest that they are supported by multiple brain systems. We next consider the brain systems involved in refixation behavior. We first review animal research and then move on to human studies from our own and other groups that point to the role of refixations in perception, attention, and memory. In the final section, we situate refixations within a general model of natural viewing behavior. We then resume our observations with the proposal that refixation behavior depends mainly on the cooperative activity of three brain systems: attentional selection of task-relevant locations guided by the dorsal attentional network, visual working memory to store the goal state in the visual cortex, and comprehension of scene meaning and higher-order task goals supported by the hippocampal memory system.

## Qualifying and quantifying refixations

Reviewing the literature on refixations is complicated by the diversity of terminology and criteria used to describe refixation behavior. We have come across the following terms used to describe this phenomenon: refixation, revisit, revisitation, reinspection, reexamining, return fixation, gaze return, repetition, regression (in reading). Importantly, these terms refer to the return of the eyes to the image location visited earlier *within* the current viewing trial. To add to the confusion, early long-term memory research sometimes used the term “refixations” to refer to the eyes returning during a test phase to locations fixated during a preceding encoding phase of the task (e.g., Foulsham & Kingstone, 2013; Holm & Mäntyla, 2007). Nowadays, the term gaze reinstatement is used to refer to those delayed gaze returns, which lead to reactivation during retrieval of encoded long-term memory representations. The neural mechanisms of gaze reinstatement have been studied repeatedly (see for a review Wynn et al., 2019). They are related to hippocampal pattern completion, which can occur hours or days after initial memory encoding. We will briefly mention gaze reinstatement, but focus on eye movement behavior within single trials, lasting several seconds of unrestricted viewing. This accords to the temporal span of visual working memory, where refixations are eye movements of a particular direction within an uninterrupted sequence of saccades and fixations. Although refixations in reading fall under this definition, we will not consider these here, because linguistic factors add another layer of complication to the dynamics of refixation control.

**Fig. 1:**
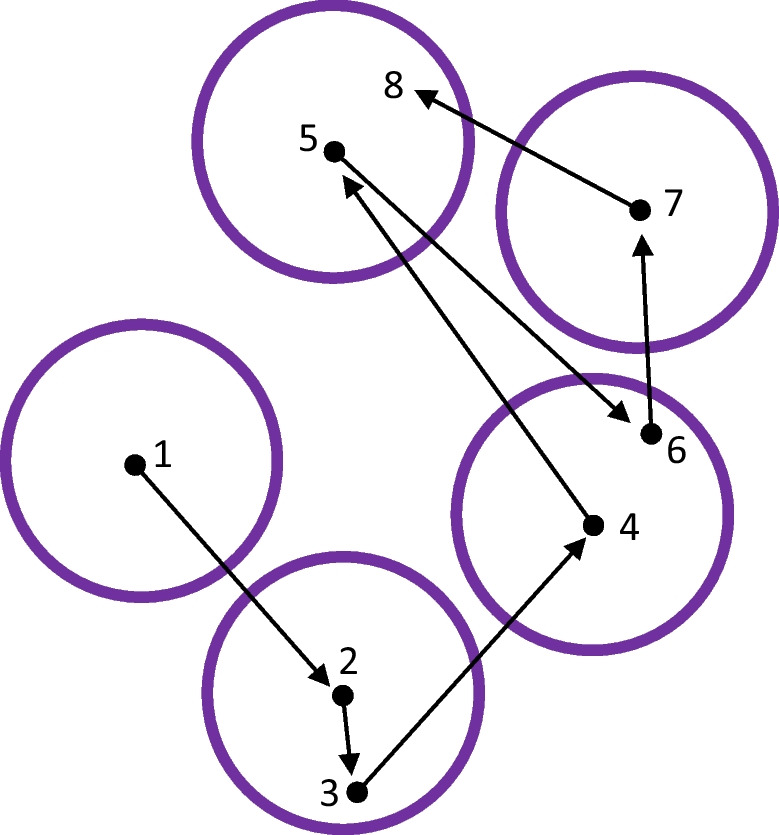
Fixation types in unrestricted viewing. A hypothetical display with five items presented in a trial where exploration involves a sequence of eight fixations (seven saccades). Numbers indicate fixation rank, the position in the order of fixations within a trial. Fixations 4 and 5 are precursor fixations. Fixation 6 is an immediate refixation (Lag-2 revisit). Fixation 8 is a nonimmediate refixation. Fixations 1, 2, 3, and 7 are ordinary fixations

Having defined refixations as returns to previously visited locations, it is then natural to define initial fixations to the locations that will be later revisited with refixations (Fig. 1). Only a handful of studies have examined these fixations, so they do not have established names. They have been called “return fixations” (Hooge et al., 2005), “fixations to return locations” (Wilming et al., 2013) and “to-be-revisited fixations” (M. Zhang et al., 2022). We focused on these initial fixations in our recent study and named them “precursor fixations” (Nikolaev, Ehinger, et al., 2023). This naming reflects their importance for understanding refixation behavior. The terminology of precursor fixations and refixations is illustrated in Fig. 1, along with the notion of refixation lags as explained in the following paragraph.

**Fig. 2:**
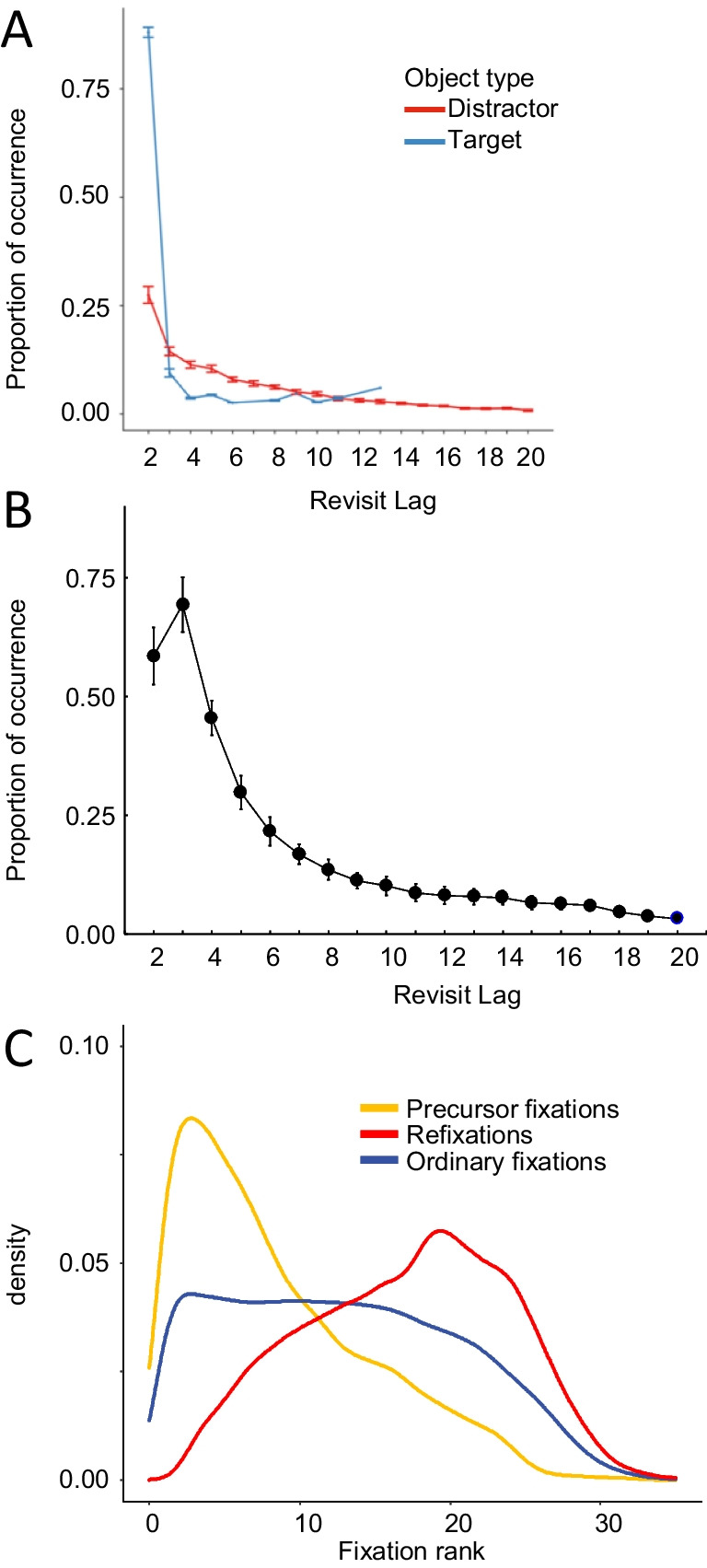
Main characteristics of refixations. **A** Proportion of objects that were revisited as a function of their object type (distractor/target) and their revisit lag (adapted from Godwin et al., 2021). **B** Proportion of revisits as a function of the lag (between precursor fixation and refixation) computed from our visual search data (Nikolaev et al., 2018). The proportion of lag-3 refixations is larger than lag-2 refixations because the 2-degree criterion for refixation selection excluded many lag-2 refixations preceded by small saccades. Vertical bars denote 0.95 confidence intervals for 21 participants. (C) Probability density estimation of precursor fixations, ordinary fixations and refixations according to the fixation rank within an 8-s trial of the visual search task in Nikolaev et al. (2018; Nikolaev, Ehinger, et al., 2023). With typically 3–4 fixations per second, the peak density of precursor fixations is within the first 2 s of viewing (adapted from Nikolaev, Ehinger, et al., 2023)

A typical question when analyzing refixations is, what should be the spatial distance between two fixations in order to treat them as related and assign to them the status of precursor fixation and refixation? This question is easy to answer when the image display consists of isolated objects on a homogeneous background. In this case, a return of gaze to the previously fixated object with some margin of tolerance can be considered a refixation. The answer is more complicated in cases of natural or artificial scenes where the distances between small elements are below the resolution of eye tracking. One possible solution is to define a certain radius around each (precursor) fixation and consider subsequent fixations that fall within this radius as refixations. In several refixation studies, this radius has been set at 2° of visual angle, referring to the approximate radius of foveal vision (Anderson et al., 2013; Gilchrist & Harvey, 2000; Nikolaev et al., 2018; Solman et al., 2011). This implies that fixations that follow the precursor fixation with small saccades are not accounted as refixations (since they do not involve spatial memory about previous fixation locations) (Fig. 2B).

Unsurprisingly, the number of precursor fixations peaks at the beginning of visual inspection, most notably within the first 2 s, while refixations tend to occur later during the trial (Fig. 2C). The interval between the precursor fixation and the refixation is an important characteristic of refixation behavior. It may provide information about the memory involved in the visual task at hand. This characteristic is known as return offset, which is the number of intervening fixations between the precursor fixation and the refixation (M. Zhang et al., 2022). The offset count is typically referred to by its lag number: immediate returns (Klein & Hilchey, 2011) are also referred to as lag-2 (Beck et al., 2006; Klein & Hilchey, 2011; Peterson et al., 2001), or lag-1 refixations, if lag is interpreted as the number of intervening fixations (McCarley et al., 2003). We will refer to them as lag-2. Refixations with larger lags are called nonimmediate refixations (Klein & Hilchey, 2011). We may distinguish them as lag-3, lag-4, and so forth.

A remarkable characteristic of refixation behavior, particularly in visual search tasks, is the very high frequency of *immediate* refixations (Klein & Hilchey, 2011) or lag-2 revisits (Beck et al., 2006; Godwin et al., 2017; Klein & Hilchey, 2011; Peterson et al., 2001). The number of nonimmediate refixations drops sharply with increasing lag (Fig. 2). The distinction between immediate and nonimmediate refixations may shed light on the role of memory in specific visual tasks. For example, immediate refixations have often been explained by a premature termination of the precursor fixation followed by a rapid realization of this error (Godwin et al., 2017; Peterson et al., 2001), whereas nonimmediate refixations may indicate reaching the capacity of working memory with accumulation of visual information in viewing behavior (Beck et al., 2006).

**Fig. 3:**
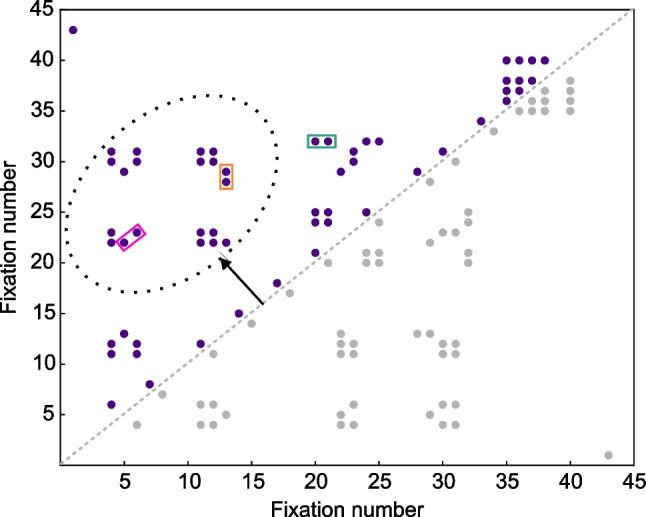
Recurrence quantification analysis of refixations. Example recurrence plot of a 10-s visual search trial. One half of this symmetric plot is used for computing the recurrence measures and is highlighted using purple dots. Fixation sequences are identified as vertical, horizontal or diagonal lines on the plot, where a line is composed of two or more dots. The pink box marks a diagonal line, indicating a revisit of locations viewed in Fixations 5 and 6 again in Fixations 22 and 23 in the same order. This repetition of a fixation sequence is quantified by the determinism measure. The orange box marks consecutive refixations (Fixations 28 and 29) on a location visited earlier (Fixation 13), while the green box marks a single refixation (Fixation 33) on a region that was viewed earlier using consecutive fixations (19 and 20). Both these clusters of visits to a location are quantified using the laminarity measure. The distance of the refixation dots from the line of symmetry, illustrated as the arrow pointing to the ellipse, indicates the time between refixations (CORM) in a trial. (adapted from Meghanathan et al., 2019). (Color figure online)

**Fig. 4:**
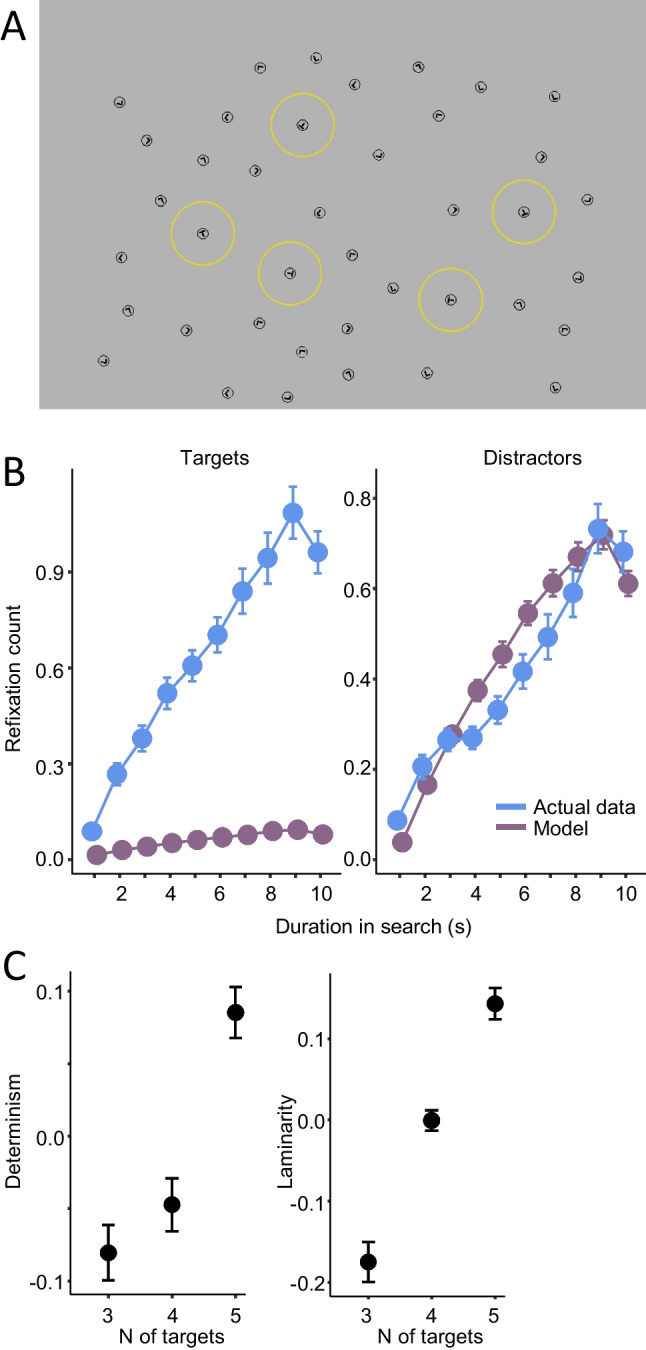
Refixations on targets and distractors in visual search. **A** Sample display used in the multitarget visual search in Meghanathan et al. (2015, 2019, 2020). Targets, which could be 3, 4, or 5 in a display, are highlighted with yellow circles (adapted from Meghanathan et al. (2020). **B** Time course of *z* scores of refixations in 1-s time windows within the 10-s search interval. The actual data is compared with a random model obtained by generating a pseudosequence of fixations for each trial display. **C** Determinism and laminarity for three, four, and five target conditions of the visual search task. (**B–C** are adapted from Meghanathan et al., 2019.) (Color figure online)

The simplest and most common approach to measure refixations is to calculate their frequency by regions and task conditions of interest. While adding a temporal dimension, refixation frequencies plotted as a function of lags does not fully capture the dynamics of refixation behavior. Anderson et al. (2013) introduced recurrence quantification analysis (RQA), a tool for quantifying periodicities in nonlinear dynamical systems, to analyze eye movement dynamics. This technique uses recurrence plots of the time series of a dynamical system’s states to indicate the moments when the system returns to a previous state. In the case of eye movements, recurrence plots summarize refixation behavior in a trial by representing the observed sequence of fixations in both *x*- and *y*-axes according to their rank (Fig. 3). RQA produces three measures of refixation behavior: determinism, which quantifies repeated gaze patterns in the recurrence plot; laminarity, which indicates the frequency of refixations to a single precursor location; and center of recurrence mass (CORM), which indicates how quickly refixations are made after precursor fixations (see the Appendix for details on RQA). RQA measures quantify differences in viewing patterns that provide insight into the memory processes at play in different tasks and displays. For example, in a scene viewing task, RQA measures depended on scene type and scene complexity and clutter (Wu et al., 2014). During a memorization task, both determinism and laminarity increased with the number of items in memory (Fig. 4C; Meghanathan et al., 2019). With increasing memory load, this result suggests, memorization increasingly comes to depend on the repetition of sequences of fixations, that is, parts of a scanpath.

## Eye-tracking insights into refixation functions

Although refixations had been studied before (e.g., Ballard et al., 1995), much interest in refixation research was spurred by the seminal Nature paper by Horowitz and Wolfe (1998), with the telling title “Visual Search Has No Memory.” One of the first subsequent attempts to study refixations was aimed at disproving the notion that visual search is a memoryless process (Gilchrist & Harvey, 2000). Since then, perhaps the majority of refixation studies have kept using visual search tasks. Depending on the type of task, refixations have been assigned several roles, each of which is somehow related to memory. Below, we loosely group refixation functions into three categories, recognizing that our list of functions is not exhaustive and that functions may overlap within a task. The first category can be called “reparative.” This term refers to the compensatory role of refixations in correcting working memory deficits caused by insufficient previous visual sampling or processing. The second category is named “constructional.” This, because the category refers to the role of refixations in the formation of representations in working memory and/or long-term memory. The third category is called “strategic,” referring to the role of refixations in managing limited memory resources for the given visual task. All these functions develop across successive eye movements, supporting the notion that memory during natural viewing changes in a highly dynamic fashion.

### Reparative functions of refixations

A prominent goal of refixations is to restore inadequate and incomplete visual processing resulting from premature interruption (Beck et al., 2006; Godwin et al., 2021; Peterson et al., 2001). This was based on the observation that shorter fixations were more likely to be followed by refixations than longer ones (Godwin et al., 2021; Hooge et al., 2005; Peterson et al., 2001; M. Zhang et al., 2022; but see Wilming et al., 2013). Interrupted fixations are particularly damaging for identification of targets. Consequently, task-relevant objects or locations receive more refixations than irrelevant ones (Wilming et al., 2013; M. Zhang et al., 2022). Participants quickly realize that they have likely missed a possible target, and as soon as they realize this, make an immediate (lag-2) refixation to the missed item (Godwin et al., 2021; Peterson et al., 2001).

Insufficient sampling of visual information at the first visit to a location is often proposed as an explanation for refixations in many different tasks. For example, insufficient sampling of numerical information was used to explain refixations in a dot counting task (Li et al., 2010). Multiple nonimmediate refixations during counting may occur due to a preliminary shift of attention away from a dot before processing is completed. Insufficient sampling was used to explain a characteristic refixation behavior in mind wandering: The scanpath becomes more repetitive and less spread out in space than when participants are focused on the (memory) task (H. Zhang et al., 2021). The authors concluded that during mind wandering, the gaze sweeps across the visual scene without detailed processing at each location. According to the authors, an increase of refixations in mind wandering may even be an adaptive response to insufficient sampling of visual information at the first visit.

Another type of memory deficit that arises in the course of natural viewing may occur because new information sampled at fixations fills working memory up to capacity. The well accepted visual working memory capacity of three to four items (Luck & Vogel, 1997) would suggest that each item should be refixated after a few intervening fixations. In reality, after peaking at lag-2, the refixation rate rapidly decreases with the number of intervening fixations (Fig. 2) and becomes lower than that of a memoryless system (Beck et al., 2006; Peterson et al., 2001). This may be a matter of forward saccadic momentum (Smith & Henderson, 2009) or IOR (Klein, 2000). But IOR does not necessarily inhibit immediate refixations (Hooge et al., 2005; Smith & Henderson, 2011), although it may reduce their frequency (Bays & Husain, 2012) or may even fail to affect refixations entirely (Shen et al., 2014). Noteworthy, IOR is predominantly observed in visual search, whereas in other tasks even facilitation of refixations is more common (Höfler et al., 2011).

McCarley et al. (2003) did observe an increase in nonimmediate refixations starting from lag-4, as expected from the accepted memory capacity. This observation was supported by Shen et al. (2014), who showed that low refixation probability is correlated with high individual memory capacity. These findings confirm the notion that a return to previous locations is needed to resample information that is no longer in (visual working) memory.

Surprisingly, some studies have even reported that the refixation rate increases again at larger lags—for example, at lag-12 (Peterson et al., 2001) or lag-9 (Beck et al., 2006), suggesting a larger capacity than typical for working memory. Fragile memory meets this requirement. It provides a rich and capacious storage (almost) like iconic memory, typically up to 12 items, while lasting as long as working memory (Sligte et al., 2008; van Moorselaar et al., 2015; Vandenbroucke et al., 2015). This memory is fragile because new information at memorized locations overwrites old information. The effect may alternatively be attributed to a high-capacity but low-resolution memory for the scanpath (Dickinson & Zelinsky, 2007; Keech & Resca, 2010). McCarley et al. (2003) proposed that participants in Peterson et al. (2001) used systematic scanning strategies that may serve as mnemonic aid (e.g., scan right to left, scan top to bottom). McCarley et al. (2003) eliminated the long lag refixations by using a gaze contingent paradigm that precluded previewing of the entire search display. However, this result is not decisive, because gaze-contingent display changes also overwrite fragile memory.

Representations in working memory can be subject to rapid decline (Henderson, 1997; Irwin, 1991). To prevent forgetting, refixations to weak object representations may provide a robust solution by serving as a *rehearsal* mechanism, as proposed by Zelinsky et al. (2011). In this elegant gaze-contingent study, participants had to remember nine real-world objects and their locations on a surface. In a subsequent test they were asked to identify the target object at a probed location. Unknown to the participants, the termination of the study display depended on the number of intervening objects viewed after the target object was fixated. Refixations of the target object increased with the number of intervening objects up to five, and this improved the accuracy in the recognition task. To model the observed refixation behavior, three components were deemed sufficient—visual working memory, IOR, and distance bias: probability of fixating an object decreases with the distance from the current fixation position. The authors concluded that refixations are made to rehearse items in working memory as their representations deteriorate.

### Constructional functions of refixations

Refixations contribute to the construction, accumulation, and updating of memory representations of objects in working memory, as well as scenes and events in episodic memory. Refixations promote the accumulation of information about object features in working memory across successive eye movements (Hollingworth & Henderson, 2002; Pertzov et al., 2009), or at least some features, as was found in a real-world experiment (Tatler, Gilchrist, et al., 2005). In this study, participants wearing a portable eye-tracker were asked to remember objects as they entered real rooms. Only object position information, but not object presence or color information, was accumulated with each refixation on the object. The authors concluded that the information accumulated during refixations updates the stored description of the object at least partially. Frequent refixations greatly increase the likelihood that a scene or an event will enter in long-term memory, presumably by repeated sampling of visual information (Kragel et al., 2021; Nikolaev, Bramão, et al., 2023), as we describe below.

The accumulation of visual information with refixations serves to support decision-making. When determining the identity or value of objects, especially complex objects in cluttered scenes, refixations help accumulate the information needed to enable the identification decision (Henderson et al., 1999; Smith & Henderson, 2009; Tatler & Vincent, 2009; Wedel et al., 2023). For example, when searching for and identifying the target coffee brand on a retail shelf, more refixations lead to more accurate search performance (Van der Lans et al., 2008). This accumulation of evidence for identification is reflected in clusters of refixations on the object in the scanpath (Wedel et al., 2023).

Accumulating visual information through refixations also facilitates decision-making in complex, multistep tasks (Spering, 2022). For example, participants visually gathered information about two gambles before choosing one of them (Manohar & Husain, 2013). Each gamble was presented on a display by two numbers: its probability and stake. Multiple refixations to these numbers accompanied the decision process. Refixations early in the trials were concentrated on numbers with the highest information gain, whereas later they were focused on those options with higher expected value. The authors explained this transition by the insufficiency of the information sampled at each fixation for making the decision. Refixations helped gather additional information by returning to locations with information relevant at a particular stage of the decision-making.

Refixations are an essential component of the scanpath in free viewing. This may be another way in which they contribute to the construction of memory. Inspired by the scanpath theory, which states that the scanpath during encoding is remembered to be repeated during recognition (Noton & Stark, 1971), multiple studies have demonstrated that episodic remembering is accompanied by scanpath replay, which assembles and reconstructs spatiotemporal scene properties (Johansson et al., 2012, 2022; Laeng et al., 2014; Wynn et al., 2016). This suggests that the scanpath may be part of memory representations. We observed the contribution of the scanpath to memory encoding in our study that used the recurrence analysis of refixations (Meghanathan et al., 2019). Participants searched during 10 s for 3, 4 or 5 target letters, *T*, in a large field of distractor letters, *L.* All letters had different orientations, and the task was to remember the orientation of the target letters (Fig. 4A). An identical test screen followed, except in one half of the trials one target had its orientation changed and participants were asked to report whether this change had occurred (Meghanathan et al., 2015). As the number of targets increased, so did the number of refixations. Recurrence quantification analysis revealed characteristic temporal patterns in refixation behavior. As the number of items in memory increased, determinism (the number of repeated fixation sequences) and laminarity (clustered fixations) also increased (Fig. 4C). Not only were precursor locations revisited, but they were revisited in the same order. Thus, parts of a scanpath are encoded for remembering items, rather than just their locations. This is essentially a corollary of the scanpath theory, indicating that repeated sequences of fixations could facilitate encoding into working memory. Note that such memory-dependent scanpath repetition is in accordance with the mnemonic function of stereotypical scanning behavior as proposed by McCarley et al. (2003).

Refixations also play a key role in the construction of robust representations in long-term memory. Increases in refixation frequency are related to successful memory formation, as evidenced by the corresponding increase in subsequent memory performance (Nikolaev, Bramão, et al., 2023; Voss et al., 2011). As we will argue below, refixations may contribute to the association of separate memory elements from multiple locations into a coherent memory representation (Kragel et al., 2021; Nikolaev, Bramão, et al., 2023).

### Strategic functions of refixations

Refixations can be used in the context of an overarching viewing strategy for memory management. Consider active sampling of visual information as akin to foraging behavior (Bella-Fernandez et al., 2022). Visual foraging involves continuous switching between the two modes of exploring new locations and exploiting previously visited ones (Bella-Fernandez et al., 2022; Gameiro et al., 2017; Wilming et al., 2013). These modes place conflicting demands on the visual system, and for optimal foraging, exploration should be balanced with exploitation.

The exploration and exploitation modes correspond to two covert states of visual attention: global and local, respectively (Antes, 1974; Liechty et al., 2003; Malem-Shinitski et al., 2020; Zangemeister et al., 1995). The alternation of these modes can be modeled as a choice between local and global attention at each fixation in a second-order Markov process (Malem-Shinitski et al., 2020). The Markov process embodies the decision rule that exploitation is performed when the salience and meaning of the current fixation is higher than that of the previous fixation. If the information gained from the current fixation is more than that of the previous fixation, exploitation continues, otherwise the viewing mode switches to exploratory.

Hidden Markov models (HMMs) have been particularly widely used to segment scanpaths into distinct phases that share similar statistical properties (Olivier et al., 2022). Refixations play a crucial role in this segmentation as one of the parameters describing latent cognitive states of the Markov model. In particular, switching between global and local covert attention within a scanpath has been described in viewing advertisements (Liechty et al., 2003), in scene viewing (Malem-Shinitski et al., 2020), and in visual search (as switching between localization and identification states; Van der Lans et al., 2008). On the other hand, the predominance of global or local attention may be an enduring style of individual behavior, reflected in holistic and analytic viewing strategies, respectively. For example, in face recognition, the holistic strategy involves looking at the center of the face, while the analytic strategy involves looking at both eyes (in addition to the face center; Chuk et al., 2014). The holistic gaze pattern is associated with intuitive judgments of emotional facial expressions involving gestalt perception, as opposed to focusing on details (Mega & Volz, 2017). The holistic strategy in learning is characterized by fewer fixations and transitions between areas of interest than the analytic strategy (Nitzan-Tamar et al., 2016). In scene viewing, the holistic strategy involves multiple switches between the foreground and background, while the analytic strategy involves looking at the foreground with less switching (Hsiao et al., 2021). Refixations are an essential component of local, analytic viewing strategy. This strategy involves scrutinizing and dwelling on an item or location, as well as revisiting already seen regions. Specifically, refixations support optimal sampling of relevant elements of a scene by matching saccade trajectories to an optimal scanpath among relevant locations on the priority map that directs eye movements (Wilming et al., 2013).

The exploration-exploitation trade-off plays out in the face of limited memory resources during visual search. In general, memory use during visual search can be measured by refixations on distractors (Hollingworth & Bahle, 2020), the rate of which determines search efficiency (Horstmann et al., 2020). Search efficiency can be manipulated, for example, by target–distractor similarity: distractors are revisited more often when searching for similar than for dissimilar targets (Horstmann et al., 2016, 2017). In these studies, distractor refixations correlated with search efficiency in the 10-item display but not in the four-item display, suggesting that refixations are involved only when memory capacity is reached.

In Meghanathan et al. (2019), target refixations kept increasing throughout the task, whereas distractor refixations were no more frequent than chance (Fig. 4B). Moreover, the number of refixations on targets increased with the number of targets in the display (3, 4, or 5), whereas the number of refixations on distractors decreased. As more targets were found, participants presumably favored revisiting already seen targets over exploring the display to avoid replacing task-relevant target information with novel distractor information in memory, thereby, drastically reducing distractor refixations.

A preferential increase in target refixations hints at a predetermined viewing strategy catering to memory capacity limits. Evidence for such a strategy was found earlier in Körner and Gilchrist (2008), where the refixation rate in target-present displays (from a target present–absent task) were compared with a one-target display (from a one-or-two targets present task) until the (first) target was found. In the one-target display, refixations of distractors increased even before the target was found indicating an early memory bottleneck. Preallocation of memory for the additional target in the one-target display limits the available memory resources for distractors, resulting in increased distractor refixations. Thus, distractor refixations are aimed at compensating for the limitation of memory capacity and its decay during visual search.

When the task involves manipulation of objects, refixating may be a viewing strategy to minimize the load of working memory. For example, refixations were measured in participants who were asked to sort bricks in a virtual environment using haptic stimulation devices (Droll & Hayhoe, 2007). The authors varied the predictability of the brick feature on which the sorting rule was based. When the task was unpredictable and the memory load was high, the refixation frequency of the bricks increased compared with a more predictable task. This indicated a switch in viewing strategy from memory based to refixation based. That is, when the elements needed to perform the task could not be fully loaded into working memory, they were refixated according to the task demands. This illustrates the principle of a trade-off between gaze and working memory use: Working memory is used in conjunction with the world, which serves as an “external memory” through gaze access of objects as needed. Furthermore, the refixation strategy may mediate the repeated comparison of perceptual information arriving at fixation with information stored in short- and long-term memory, which underlies goal-directed visual exploration (Hollingworth & Henderson, 2002; Pollmann & Schneider, 2022)

To be part of an effective viewing strategy, refixations should be pre-programmed in advance in a goal-dependent manner. This raises the question, could a queue of potential saccade targets include refixations? Some indications come from research on the parallel programming of saccades. Parallel programming (i.e., simultaneous preparation of motor programs for several saccades; Becker & Jurgens, 1979), has been reported for simple saccade-target tasks (Findlay et al., 2001; McPeek et al., 2000; McSorley et al., 2020), as well as during free viewing of scenes or real-world objects (Wu et al., 2013, 2016). The reported length of the planned scanpath is limited to a sequence of two to three saccades (De Vries et al., 2014; Hoppe & Rothkopf, 2019). Such a short length, however, does not preclude these short viewing plans from containing repetitive destinations. This is suggested by the absence of IOR in parallel saccade sequences (MacInnes et al., 2015). This study measured the speed of eye movement responses to probes appearing at previously fixated locations when these locations were visited in both parallel and independent saccade sequences. IOR was observed in independent but not parallel sequences. Furthermore, IOR was reduced at an intermediate location within the parallel sequence, opening the possibility for refixation. However, to our knowledge, no study directly indicates the presence of refixations in parallel saccadic sequences.

It should also be noted that the refixation strategy is not always effective in minimizing memory usage. For example, when it is necessary to hold several targets in memory in order to perform hybrid search, which combines visual and memory search (Drew et al., 2017). In this study, participants searched for one of multiple memorized targets in a visual display consisting of either 8 or 16 items. The dwell time on each distractor increased with the memory set size, suggesting that a memory search was performed each time a new distractor was fixated. The number of distractor refixations also increased with the set size. But this increase was too small to indicate a strategy involving repeated visual searches for target subgroups. Thus, hybrid search follows the strategy of “one visual search, many memory searches,” which discourages refixations.

To summarize, eye-tracking studies revealed three primary categories of refixation functions. Reparative refixations serve to rectify deficiencies in information sampling or processing, particularly at target locations. Refixations aid in compensating for memory deficits that arise from reaching the limits of memory capacity during demanding tasks, and assist in recovering or rehearsing information that has been lost due to forgetting. Constructional refixations contribute to updating of the stored representations, facilitating the accumulation of visual feature information. Refixations become part of the scanpath, which to some extent is incorporated into memory representations, thereby supporting their retrieval. By increasing visual sampling, refixations contribute to the creation of robust memory representations and help bind separate locations into a coherent memory. Strategic refixations contribute to visual sampling by switching from a mode of exploring new areas to exploiting previously visited ones. In interactive tasks, the refixation strategy minimizes the use of working memory by relying on the external environment as an extended memory system that can supply the necessary information when gaze returns to specific locations.

Eye-tracking studies have revealed a wide range of roles that refixations can play in goal-directed visual behavior. Knowledge about the neural mechanisms of the refixation functions is currently much more limited and concerns mainly the mechanisms of refixation guidance and the contribution of refixations to memory formation, which we discuss in the next section.

## Neurophysiology of refixation behavior

### Brain structures involved in refixation-related functions

**Fig. 5:**
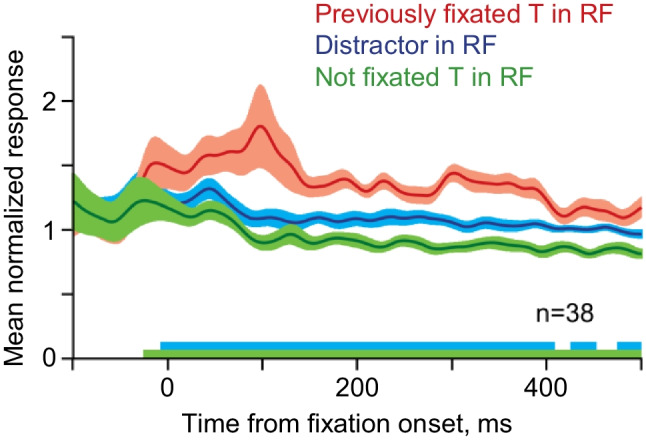
FEF neurons keep track of fixated items. The mean normalized responses of the population of 38 putative tracking neurons aligned by fixation onset when a target (T) that had been fixated previously in the trial (i.e., refixated) was in the receptive field (RF), when a distractor was in the receptive field, and when a target that had not been fixated earlier was in the receptive field. The width of the traces indicates the *SEM*, where *n* indicates the number of neurons contributing to the trace. The lines along the *x*-axis indicate times at which the spike-density functions of the fixated target and distractor (blue line) or the not fixated target and the fixated target (green line) were significantly different (adapted from Mirpour et al., 2019). (Color figure online)

Information about brain structures related to refixation behavior is rather scarce. To our knowledge, a direct relationship with refixations has so far been established for two structures: the frontal eye field (FEF) and hippocampus. Specialized neurons in the FEF keep track of items that have been fixated within a trial (Mirpour et al., 2019). Two macaque monkeys were trained to find multiple targets among distractors in free-viewing visual search, while the activity of neurons in the FEF was recorded. Activity in 38 of the 231 recorded neurons was increased when the search item was previously fixated within the same trial (Fig. 5).

These neurons differed from other FEF neurons in that their activity was not suppressed during fixation, had no motor components, and remained constant throughout the trial. The authors concluded that these neurons monitor previously fixated items and send an inhibitory tagging signal to the lateral intraparietal area (LIP), which drives IOR (Mirpour et al., 2009). Beside LIP and FEF (Bichot & Schall, 2002; Klein, 2000; Mayer et al., 2004), IOR was found to be associated with the superior colliculus (Posner et al., 1985; Taylor & Klein, 1998).

Remarkably, these three structures exactly are considered together to compose a priority map that guides eye movements (Bisley & Goldberg, 2010; Bisley & Mirpour, 2019), including refixations. Furthermore, the hippocampus plays a special role in refixation control by providing online memory representations to the oculomotor regions that guide visual exploration (Voss et al., 2017). As we describe in detail below, hippocampal theta activity drives revisits of previous locations, as evidenced by the theta modulation just prior to refixation (Kragel et al., 2021).

In natural viewing, the synergistic activity of FEF neuronal ensembles encodes both visual stimuli and eye movements across the entire visual field (Dehaqani et al., 2018; Khanna et al., 2019). This allows FEF to specify a series of targets for future saccades (Phillips & Segraves, 2010). Moreover, the FEF plays an important role in visual attention by showing selectivity for target features to locate the target in an array of distractors, i.e., discriminating between a target and distractors (Bichot et al., 1996, 2015). Considering these FEF functions, the existence of FEF neurons monitoring previously fixated items, as well as the dense structural interconnections of FEF with the hippocampus (Ryan et al., 2020; Shen et al., 2016), it is likely that the cooperation of these brain structures is important for refixation control. In tandem, the hippocampus provides online memory representations to FEF, which directly controls oculomotor behavior and directs ongoing exploratory viewing (Voss et al., 2017). However, these neural mechanisms do not suffice, in particular, to account for the dependency of refixations on working memory.

### Refixation research using EEG–eye-movement coregistration in free viewing

In humans, although eye-tracking studies have described refixation behavior in great detail, these studies face limitations in explaining its neural mechanisms. The temporal scale of natural eye movements of several hundred milliseconds dictates that the appropriate methods to study the associated brain activity should have a high temporal resolution, such as EEG or MEG. However, this is where the major problem of free-viewing brain activity research comes in. In natural viewing, sequential eye movements produce systematic effects on EEG or MEG (Dimigen et al., 2011; Nikolaev et al., 2016). Each saccade evokes a brain response that overlaps with the responses to the previous saccades because the intervals between saccades are too short (200–300 ms) to allow time for the responses to fade out. Given the linear summation of electrical fields (Nunez & Srinivasan, 2006), the resulting EEG waveform is actually a sum of overlapping responses. Moreover, the low-level oculomotor properties of eye movements, such as saccade size or direction, influence the saccade-evoked responses. These eye movement effects can be confounded with the effects of the experimental conditions of interest, which often have a characteristic gaze pattern. Importantly, these effects are distinct from oculomotor artifacts, the elimination of which has long been the focus of much research (Gratton et al., 1983; Lins et al., 1993). There are three major classes of oculomotor artifacts: those due to eyelid movement (blinks), rotation of an eyeball, and eye muscle contraction (saccadic spike activity). Nowadays, all their effects on the brain activity can successfully be removed thanks to the development of artifact correction procedures based on blind-source separation, such as Independent Component Analysis (ICA; Jung et al., 2000). Some of these procedures are specifically sharpened to remove oculomotor artifacts in free viewing, achieving high efficiency in eliminating them (Dimigen, 2020; Plöchl et al., 2012). However, these procedures cannot remove the overlapping or low-level effects of eye movements, which are difficult to separate due to the intercorrelation of fixation duration, saccade size, and direction (Tatler & Vincent, 2008).

Over the past decade, several solutions have been proposed to the problem of overlapping saccadic responses. One solution consists in matching of eye-movement characteristics between experimental conditions (Devillez et al., 2015; Dias et al., 2013; Dimigen et al., 2011; Fischer et al., 2013; Kamienkowski et al., 2012; Nikolaev et al., 2016). This solution does not eliminate the overlap effect, but after matching, their effects on brain activity are presumed to be equal and hence no longer confound comparisons between conditions. The question is which eye-movement characteristics need to be matched (e.g., fixation duration, saccade size, or direction). Since several of them often differ at the same time, methods have been developed to match multiple eye-movement characteristics in multidimensional covariance space. One of them involved calculating the Mahalanobis distance between the center of this space and the characteristics of each data point for two or more conditions (Dias et al., 2013; Nikolaev et al., 2016). Then, in an iterative procedure, a threshold is set for the distance, and eye movement characteristics above the threshold are excluded from further analysis. The difference between conditions is than estimated again for the remaining characteristics. If the difference still exists, the threshold is lowered and a further set of eye movements are excluded. This process continues until there is no difference in eye movement characteristics between conditions. Finally, fixation-related EEG epochs corresponding to the fixation intervals with matched eye movements are selected.

Matching provides an unbiased comparison of fixation-related EEG between conditions, but at the cost of losing data by excluding unmatched EEG epochs. In addition, in many free-viewing tasks, eye movements differ systematically between experimental conditions. Although matching excludes epochs with eye movements that differ between conditions, this technique may also eliminate epochs with characteristic gaze patterns differentially related to perceptual or cognitive processing between conditions and are therefore of research interest.

Instead of selecting epochs with the same eye movements, an alternative solution is to statistically correct for overlapping effects with regression-based deconvolution modeling (Cornelissen et al., 2019; Dimigen & Ehinger, 2021; Guérin-Dugué et al., 2018; Kristensen et al., 2017; Litvak et al., 2013). In this approach, the overlapping fixation-related EEG activity is treated as a linear convolution of fixation latencies with unknown isolated EEG responses. Deconvolution recovers these isolated EEG responses given the continuously measured EEG and fixation latencies. Such correction using linear regression still does not account for the nonlinear effects of oculomotor effects of eye movements, such as saccade size on EEG (Dandekar et al., 2012; Ries et al., 2018). However, deconvolution can be combined with generalized additive modeling (GAM; Wood, 2017), which allows modeling the nonlinear dependencies of EEG on eye movement parameters as nonlinear splines (Van Humbeeck et al., 2018). Such a solution is provided by the Unfold toolbox for MATLAB (Ehinger & Dimigen, 2019). The results of deconvolution modeling are beta coefficients representing partial effects of the predictors of interest. Thus, the output of deconvolution corresponds to averaged EEG activity similar to event-related potentials, unlike the output of the matching procedure, which consists of single EEG trials.

An important decision in the coregistration analysis is which moment to choose as the time-locking event for EEG segmentation: saccade onset or fixation onset. Although the resulting waveforms have much in common, these modes of segmentation capitalize on different types of brain processes (Nikolaev et al., 2016). Segmentation relative to saccade onset is indicative of saccade planning, which occurs in the presaccadic interval. During planning, the target of the next saccade is selected by shifting attention to the next location prior to saccade execution (Deubel & Schneider, 1996; Hoffman & Subramaniam, 1995). The attentional shift is reflected in the presaccadic EEG activity over parieto-occipital areas (Gutteling et al., 2010; Kovalenko & Busch, 2016; Krebs et al., 2012; Nikolaev et al., 2013; Ptak et al., 2011; Wauschkuhn et al., 1998).

Segmentation relative to fixation onset is indicative of visual perception at fixation in the postsaccadic interval. The evoked activity in this interval is characterized by the lambda wave over the occipital areas about 100 ms after the fixation onset, which is a response of the visual cortex to the shift of the retinal image and reflects early perceptual processes at fixation onset (Dimigen et al., 2009; Kazai & Yagi, 1999; Ossandón et al., 2010; Thickbroom et al., 1991). Subsequent evoked components (e.g., P3) are usually treated as analogues of those in ERP research (Kamienkowski et al., 2012). The choice of saccade onset or fixation onset as the time-locking event depends on the purpose of the study. Both segmentation approaches may shed light on the neural mechanisms of refixation behavior, as we describe below.

When analyzing neural activity associated with refixations, it is crucial to consider a specific factor that affects fixation-related EEG results in particular. Fixation rank, the order of a fixation within a trial, alone affects fixation-related EEG (Fischer et al., 2013; Guérin-Dugué et al., 2018; Kamienkowski et al., 2018). This poses a problem, as refixations by definition occur later during a trial than their precursors, and also tend to occur later than the ordinary fixations typically used as a reference. The problem can be solved by introducing a control analysis with *mock* pairs of precursor fixations and refixations. The fixations in the mock pairs should have the same fixation orders and lags as the actual precursor fixations and refixations, but should otherwise be unrelated to each other (Nikolaev et al., 2018). Alternatively, fixation rank can be considered by including it as a covariate in the deconvolution model (Nikolaev, Bramão, et al., 2023; Nikolaev, Ehinger, et al., 2023). Accounting for fixation rank is essential in order to determine whether the observed EEG effects associated with refixations reflect processes specific to the accumulation of visual information over time.

### Neural mechanisms of refixation planning and visual perception at refixations and precursor fixations

**Fig. 6:**
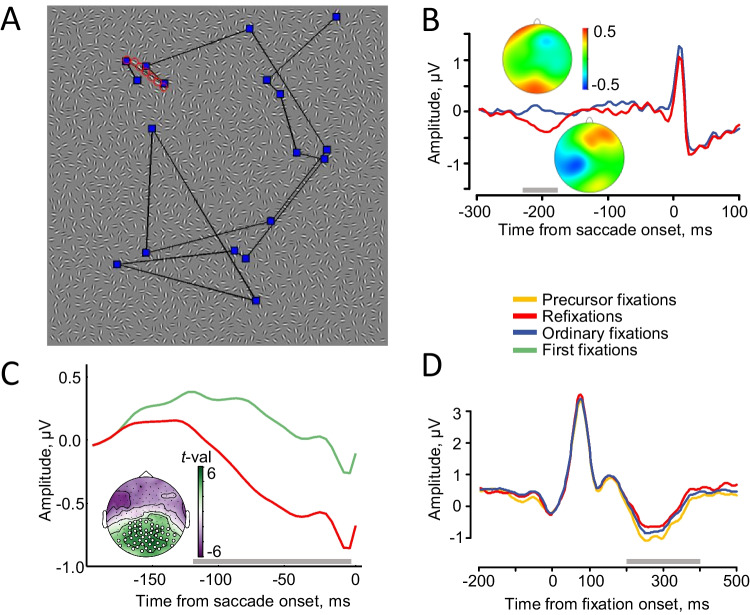
Saccade- and fixation-related potentials related to refixation behavior in visual search tasks. **A** A field of randomly oriented Gabor elements used in the visual search for ‘a snake’, a contour of seven collinear elements in (Nikolaev et al., 2018; Nikolaev, Ehinger, et al., 2023). **B** Grand-averaged presaccadic potential for the left parietal area baseline-corrected at −300–280 ms from saccade onset. Map insets show amplitude distribution at −200 ms for ordinary fixations (top) and refixations (bottom). (Adapted from Nikolaev et al., 2018). **C** Grand-averaged presaccadic potential for the occipital cluster baseline-corrected at −180–160 ms from saccade onset. The green bar along the *x*-axis indicates the interval of significant difference between first fixations and refixations. The difference exists for targets, but not for distractors. Map inset shows the topography of *t* values averaged in the interval indicated by the green bar in the occipital cluster. (Reprinted from Meghanathan et al., 2020, with permission from Elsevier). **D** Grand-averaged postsaccadic potential (regression betas obtained after deconvolution) for the right occipital area baseline-corrected at 0–20 ms from fixation onset. (Adapted from Nikolaev, Ehinger, et al., 2023). The grey bars above the *x*-axis indicate intervals of significant difference. (Color figure online)

Our first study of refixation behavior in humans was exploratory and thus used both types of time-locking events (Nikolaev et al., 2018). We analyzed EEG related to refixations in a free-viewing task, which involves visual search for a contour of seven collinear Gabor elements (‘a snake’) in a dense field of small Gabor elements of random orientation. Our previous analysis of this dataset revealed increased EEG amplitude before saccades to nonsalient locations, suggesting that greater attentional effort is required to select less salient saccade targets (Van Humbeeck et al., 2018). Due to a multitude of spurious stimulus groupings, visual search in this task is quite challenging, and we expected many refixations during an 8-s search trial (Fig. 6A). Indeed, about 13% of the eye movements during contour search were refixations. We hypothesized that refixations differ from ordinary fixations in saccade planning and extracted the presaccadic potential by time-locking EEG epochs to saccade onset. Alternatively, refixations may differ in the sampling of visual information at the revisited location, as refixation may involve repetition suppression (Epstein et al., 2008) and/or updating of previous memory representations (Gilchrist & Harvey, 2000; Tatler, Gilchrist, et al., 2005; Zelinsky et al., 2011). Therefore, we also extracted the postsaccadic potential time-locked to fixation onset. We found that refixations were characterized by a greater negative amplitude than ordinary fixations over the left centro-posterior areas, about 200 ms before the saccade onset (Fig. 6B). This modulation of the presaccadic potential indicates that refixations differ in the allocation of attention to the next saccade target. This is evidence for distinct saccade planning for refixations, which is related to the amount of attention directed to revisited locations.

The preceding analysis did not consider the task-relevance of refixations—that is, we did not analyze refixations to the search target (a contour of collinear elements), because, after finding the contour, participants actually stopped searching. In a follow-up study, we asked how saccade planning in refixation behavior is influenced by top-down factors such as the relevance of fixation locations to the visual task (Meghanathan et al., 2020). This involved the analysis of EEG coregistered with eye movements in the study by Meghanathan et al. (2015, 2019), described above. In that experiment, we asked participants to detect and remember the identity and location of multiple targets in anticipation of a change detection test. We analyzed only the first, search stage of the task, where first fixations and refixations were considered separately for targets and distractors. To investigate the effect of task-relevance on saccade planning, we compared the amplitude of the presaccadic potential between first fixations and refixations (matched for saccade size and fixation duration) for targets and distractors separately.

We found a prominent difference in the presaccadic amplitude for targets (Fig. 6C) but not for distractors. Thus, the distinct saccade planning of refixations that was found in the first study appears to occur only when the revisited items are task-relevant. The results of these two studies suggest that the particular shift of momentary attention observed for revisited locations can be boosted by the top-down allocation of attention when these locations are relevant (targets). This allows for prioritized selection of target locations for refixations. Prioritization may facilitate the acquisition of visual information (e.g., to compensate for a memory deficit), consistent with the reparative refixation function.

Having established the neural mechanisms of refixation planning, we examined the neural activity at precursor fixations, fixations at locations that are subsequently revisited (Nikolaev, Ehinger, et al., 2023). Previous eye-tracking studies have been inconsistent regarding the characteristics of precursor fixations. One study reported that precursor fixations were 20 ms shorter than ordinary fixations and that the difference in duration between precursor and ordinary fixations was positively correlated with refixation rate (Hooge et al., 2005). In contrast, Wilming et al. (2013) found that precursor fixation durations were longer than ordinary fixations. They explained this discrepancy by their observation that refixated locations are more salient than others, implying that these locations require more scrutiny. We hypothesized that distinctive sampling of visual information on precursor fixation locations instigates their revisits. We reanalyzed the contour search dataset used in the first refixation study (Nikolaev et al., 2018), this time distinguishing postsaccadic activity time-locked to the onset of precursor fixations, refixations, and ordinary fixations that were neither precursors nor refixations. To deal with the overlapping effects of unrestricted eye movements on EEG, we here used deconvolution modeling (Ehinger & Dimigen, 2019).

Precursor fixations were found to be characterized by the largest size of the incoming saccade and the smallest size of the outgoing saccade among the three fixation categories. We interpreted this change in saccade size as evidence for a shift from an exploratory, holistic mode of viewing to an exploitative, analytic mode. The exploratory viewing mode involves a broad scanning of new locations in a scene, whereas the exploitative mode involves scrutinizing visual details and refixating previously visited locations (Gameiro et al., 2017).

In the postsaccadic potentials, we found no difference in lambda wave amplitude between precursors fixations, ordinary fixations and refixations. However, in the late 200–400-ms interval of the postsaccadic potentials, we found a more negative amplitude over the occipital areas for precursor fixations compared with both ordinary fixations and refixations, which did not differ from each other (Fig. 6D). The precursor amplitude difference was predominant over the occipital areas, suggesting involvement of the visual cortex. The early visual areas support representation and accuracy of information in short-term memory (Emrich et al., 2013; Hallenbeck et al., 2021; Rademaker et al., 2019). The precursor amplitude difference may thus be associated with visual working memory activity needed to memorize locations in order to return to them later.

In sum, our studies on the immediate saccade guidance leading to refixations indicate that a distinct attentional selection of the next saccade target occurs before refixations, but only when refixations are task relevant. Information sampling at refixations does not differ from the ordinary fixations, but it does differ from precursor fixations. This suggests that precursor fixation locations serve as pivotal moments in visual exploration and may be instrumental in the formation and updating of a viewing plan. This plan may be part of an efficient viewing strategy, in accordance with the strategic function of refixations.

### Neural mechanisms of refixations in long-term memory

**Fig. 7:**
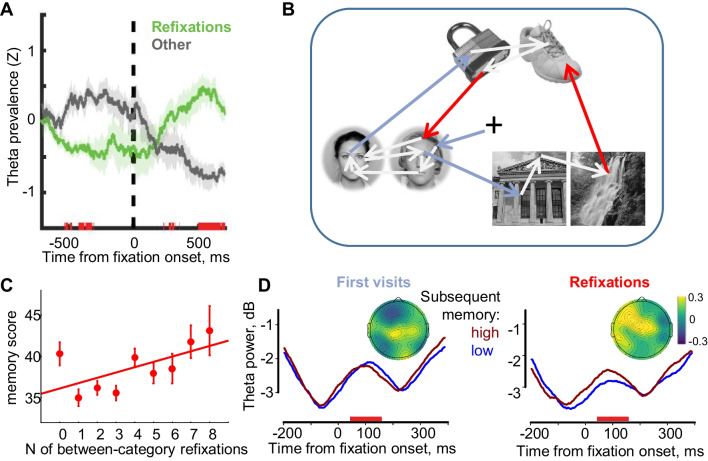
Neuronal and oscillatory activity associated with refixations. **A** Hippocampal theta activity (i.e., prevalence of oscillations) relative to the onset of refixations and other fixations. (From Kragel et al., 2021. ©The Authors, some rights reserved; exclusive licensee AAAS. Distributed under a CC BY-NC 4.0 license. Reprinted with permission from AAAS. The label ‘Revisitations’ is replaced by ‘Refixations’.) **B** Example of an encoding display (the people whose faces are shown have consented to the publication of their faces) with the scanpath overlaid. The color arrows illustrate a subset of task-relevant, between-category first visits (blue) and refixations (red) preceding the fixation intervals used in the EEG analysis. The grey arrows are saccades not included in this analysis. **C** Subsequent memory performance increases with the number of task-relevant refixations. **D** Theta power over the left frontal area and power difference maps (high minus low memory conditions) for first visits and refixations (**B–D** are adapted from Nikolaev, Bramão, et al., 2023). The red bars above the *x*-axis indicate intervals of significant difference. (Color figure online)

Refixation behavior plays a pivotal role in long-term memory, supporting the formation of long-term memory representations (Kragel et al., 2021; Kragel & Voss, 2022; Nikolaev, Bramão, et al., 2023). The neural mechanism underlying this process is reflected in brain activity in the theta EEG frequency band between 4 and 8 Hz. Specifically, refixations are predicted by the theta activity of the hippocampus, which differs in the presaccadic interval, depending on whether the next saccade is made to the previously visited or a new location (Kragel et al., 2021). In this study, intracranial EEG was recorded from several brain structures including the hippocampus of six participants (epileptic patients awaiting surgery), while they were asked to remember photos of natural scenes in anticipation of a recognition test (an old/new task). During memory encoding, participants frequently refixated previously visited locations. In contrast to other fixations, these refixations (they used the term “revisitations”) predicted the repetition of the encoding gaze pattern during the test stage of the experiment (gaze reinstatement); that is, were beneficial for the formation of long-term memory for scenes. Hippocampal theta activity time-locked to the fixation onset showed reciprocal relationships for refixations and other fixations. For refixations, theta oscillations decreased in the presaccadic interval, indicating retrieval, and increased in the postsaccadic interval, indicating encoding of the revisited locations. The opposite pattern was observed for other fixations (Fig. 7A). Thus, within an encoding trial, hippocampal theta predicted whether the next saccade would direct the eye to the previous or new location. The authors concluded that refixations are temporal markers of when short-term memory retrieval guides eye movements. Furthermore, the authors proposed that the elevated theta activity during refixations reflects integration of visual information sampled across multiple fixations with a hippocampal representation of the scene, which is stored in long-term memory.

The relationship between theta activity during refixations and the association of visual elements was also found in our recent study of episodic memory formation across eye movements coregistered with scalp EEG (Nikolaev, Bramão, et al., 2023). Healthy participants were asked to remember nine displays representing episodic memory events that were presented sequentially for 10 s. Each event consisted of two exemplars from three categories (faces, places, objects), positioned so that exemplars from the same category were close to each other, while the categories were more distant (Fig. 7B). After a distractor task, memory was tested only for the between-category associations of each event. This allowed us to separate the between-category saccades that were important for these associations, and thus enabled participants to succeed in the memory test, from the between-exemplar saccades that were less important. We divided both saccade types into those leading to first fixations and those leading to refixations.

In the subsequent memory test, performance increased with the number of task-relevant, between-category refixations (Fig. 7C). We used deconvolution to account for the overlapping effects of eye movements on the EEG (Ehinger & Dimigen, 2019). The number of between-category refixations predicted subsequent memory performance for the entire event. Theta activity accompanying these between-category refixations was increased for high compared with low subsequent memory (Fig. 7D). However, theta activity did not differ between the first between-category fixations and between the first fixations and refixations after between-exemplar saccades. Since refixations between categories were crucial for the association of event elements, the observed theta effect may reflect binding of the elements into coherent episodic memories. Thus, our findings with scalp-recorded EEG strongly support the intracranial EEG results on the key role of refixations in the construction of long-term memory representations (Kragel et al., 2021).

Taken together, the findings from the unrestricted viewing experiments confirm the findings of a body of previous studies in which stimuli were presented at fixations and eye movements were not allowed, and point to the importance of the hippocampal theta system for binding representations of long-term, episodic memory (Hanslmayr et al., 2016).

In sum, refixations during free viewing can indicate moments when the oculomotor system seeks confirmation of the relevance of a location by querying it in short- and long-term memory (Kragel & Voss, 2022). This leads to increased sampling of information from that location, which contributes to the formation of representations in long-term memory. This way, refixations fulfill their constructional role.

## Towards a neural model of refixation behavior

Returning the gaze to a previously visited location may appear a simple oculomotor act. The apparent simplicity, however, belies the extremely variegated nature of refixation behavior. Such behavior reflects multiple perceptual and cognitive functions strongly depending on the task, and involves multiple neural mechanisms supported by multiple brain structures. These complexities notwithstanding, could refixations be incorporated into an existing saccade guidance model? M. Zhang et al. (2022) recently presented a model that successfully generates empirically plausible eye movement sequences. The model is based on a winner-take-all mechanism, which takes into account image salience maps, target similarity maps, saccade size constraints and IOR. The model predicts refixation patterns as observed in a variety of visual tasks. But it reduces refixations to random events, and is uninformative with respect to the role of refixation in memory.

Refixations, as argued, may play reparative, constructional, and strategic roles in memory. Supporting the interaction between short- and long-term memory as well as the momentary alternation between memory encoding and retrieval during visual exploration (Kragel & Voss, 2022), refixations reflect the dynamics of memory across eye movements. To arrive at a more elaborate picture of memory-influenced natural viewing, we incorporate refixation behavior into a synthesis of leading saccade guidance models (Gameiro et al., 2017; Henderson et al., 2019; Võ et al., 2019; Võ & Henderson, 2010; Wilming et al., 2013; Zelinsky & Bisley, 2015). In a novel addition, we propose a high-capacity, low-resolution viewing plan that interacts with the priority map. Furthermore, we propose three candidate brain structures that, together, control and execute refixations: the dorsal attention network (DAN), the visual cortex, and the hippocampus. Specifically, these structures control saccade execution, immediate saccade planning with selective shifts of attention, tracking of both relevant and irrelevant locations, storage of accumulated information in short-term memory, and continuous exchange of information with long-term memory. Below we elaborate on how refixations can be realized through the coordinated activity of these brain structures.

### Refixations on a priority map in the dorsal attention network

Scene viewing occurs in three major stages: a scene onset response with a central bias, an exploratory stage with dispersed fixations across the scene, and a final stage with cyclic exploratory-exploitative fixations (Gameiro et al., 2017; Schütt et al., 2019; Tatler & Vincent, 2008; Wilming et al., 2013).

When a visual scene appears, it takes a saccadic latency of about 200 ms from its onset to the initiation of the first saccade (Carpenter, 1988; Schütt et al., 2019). In this short time period, a priority map containing the distribution of potentially relevant locations is generated. These locations constitute targets for eye movements (Võ & Henderson, 2010; Zelinsky & Bisley, 2015). The priority map is obtained by a combination of bottom-up scene features and top-down influences. The bottom-up features provide a saliency map of low-level features such as luminance, color, contrast, orientation, and spatial frequency (Itti & Koch, 2001). Top-down influences include contextual information (Oliva & Torralba, 2006) and scene grammar (Võ et al., 2019) retrieved from long-term memory. A quantitative meaning map is likely constructed representing the semantic values of scene regions (Henderson et al., 2019). Top-down influences also include task goals. These goals form a target map, which indicates the similarity of each location to the target in the case of visual search tasks (Zelinsky & Bisley, 2015). We propose that top-down goals also incorporate the viewing strategy. As discussed earlier, mnemonic strategies could be used to view a scene (Gilchrist & Harvey, 2006). For example, a scene could be progressively viewed in a clockwise direction or in a reading-like fashion (Tatler, Baddeley, et al., 2005), each of which strategies could be incorporated as a directional bias in a priority map. In visual search, a systematic component was found in scanpaths containing 10–20 fixations (Gilchrist & Harvey, 2006).

With all these sources combined into the priority map, the next fixation location is selected as the maximum value determined by a winner-take-all mechanism. An exponentially decaying memory function and an opposing linearly decaying IOR function (Zelinsky et al., 2011) are also incorporated into the priority map, such that a fixation on a location resets both memory and IOR values. A dip in memory for a location below a threshold, that is, ‘forgetting’, would increase the likelihood of a refixation to the location, whereas the IOR would have the opposite effect.

Subsequent saccadic exploration is characterized by a gradual widening of the fixation density from the center of the scene, that is, fixations become more distributed across the scene (Schütt et al., 2019) through larger saccades and shorter fixations. This gaze pattern can be triggered, not only by the sudden appearance of a visual scene (Pannasch et al., 2008; Unema et al., 2005), but also by scene cuts in a movie (Pannasch, 2014), by subjective boundaries of episodic events in a perceptual event segmentation task (Eisenberg & Zacks, 2016), and even without external visual changes, by switching between different subtasks in a multistage task (Guo et al., 2022). The initial exploration lasts about the first 2 seconds (Pannasch et al., 2008; Unema et al., 2005). During exploration, locations are visited that were designated as visually or semantically salient at scene presentation. If such a location is found to be task-relevant, we propose that its activation in the priority map is increased, marking it as a precursor for a later revisit (Nikolaev, Ehinger, et al., 2023; M. Zhang et al., 2022). A dynamic viewing plan or strategy is thus implemented in the form of a priority map that is updated at each fixation based on the local values in the component maps (salience, meaning, target, IOR, memory decay) such that the next saccade is directed to the location with highest priority according to the updated priority map. Refixations are relatively rare during this initial stage of scene exploration (Fig. 2C).

Subsequent viewing, driven by the priority map, uncovers the scene details necessary to achieve the visual (task) goals. Periods of exploration trade-off with periods of exploitation (Gameiro et al., 2017; Tatler & Vincent, 2008; Wilming et al., 2013) in a cyclical manner (Yarbus, 1967). The trade-off can be understood as an alternation between global/holistic and local/analytic attentional states, driven by a Markov process that probabilistically configures the scanpath (Chuk et al., 2014; Liechty et al., 2003; Malem-Shinitski et al., 2020; Olivier et al., 2022). In this trade off, precursor fixations play a pivotal role. Precursor fixation locations tend to be more salient or task-relevant compared with other locations (Wilming et al., 2013; M. Zhang et al., 2022). They are characterized by large incoming saccades and small outgoing saccades (Nikolaev, Ehinger, et al., 2023), indicating a shift from an exploratory to an exploitative mode of viewing. In exploitation, gaze visits to relevant locations are characterized by long fixations, sequences of small saccades (Pannasch et al., 2008; Tatler & Vincent, 2008; Unema et al., 2005), as well as refixations (Wilming et al., 2013).

To discourage the eyes from going to locations that have previously been visited, the priority map incorporates IOR (Zelinsky & Bisley, 2015; Zelinsky et al., 2011). But the IOR is not sufficient to prevent a high frequency of immediate (lag-2) refixations (Godwin et al., 2017). Immediate refixations of the higher priority precursor locations occur because of their higher salience compared with ordinary locations (Wilming et al., 2013; M. Zhang et al., 2022), higher similarity to targets, as evidenced by the tendency to refixate target locations (Peterson et al., 2001).

The priority maps are likely to be located in the dorsal attention network (DAN), which consists of the FEF and the intraparietal sulcus (IPS; Silver & Kastner, 2009; Zelinsky & Bisley, 2015). The FEF directs attention across the entire visual field (Dehaqani et al., 2018; Khanna et al., 2019) and discriminates targets from distractors within the visual field (Mirpour et al., 2009). Similarly, presaccadic attentional selection of task-relevant items supports the control of immediate refixations (Meghanathan et al., 2020; Nikolaev et al., 2018). Most importantly, the FEF contains specialized neurons that keep track of items being fixated within the trial (Mirpour et al., 2019). Activity in these neurons is indicative of the memory decay component of the dynamic priority map. Another part of the DAN, LIP, the monkey’s analog of human IPS, is also found to discriminate between targets and distractors (Mirpour et al., 2009). Moreover, target regions that were visited earlier and, therefore, are not relevant to the current task goals show lesser activity on the priority map. Both FEF and IPS are involved in IOR (Klein, 2000; Mayer et al., 2004; Mirpour et al., 2009). Thus, both FEF and IPS contain task-relevant priority maps that determine the execution of immediate refixations. This suggests that the DAN is the primary candidate structure underlying refixation behavior.

### Storing task-relevant locations for later revisits in the visual cortex

We further consider the possibility that refixations occur according to a strategic viewing plan. As mentioned above, an overarching viewing strategy may involve an initial sequence of fixations on a scene to identify locations that are potentially important for task completion. These precursor fixation locations are stored in memory as future refixation targets (Hooge et al., 2005; Nikolaev, Ehinger, et al., 2023; Wilming et al., 2013; M. Zhang et al., 2022). The ‘plan’ consisting of these locations may be stored in long-range, fragile location-specific memory with high capacity but low resolution (Dickinson & Zelinsky, 2007; Keech & Resca, 2010; Peterson et al., 2001; Sligte et al., 2008; van Moorselaar et al., 2015; Vandenbroucke et al., 2015). In a memorization task, the viewing plan guides repetition of scanpath sequences (Meghanathan et al., 2019). In the case of visual search, the viewing plan guides nonimmediate refixations, which occur later, with a lag up to 9–12 items (Beck et al., 2006; Keech & Resca, 2010; Peterson et al., 2001; Fig. 2A). However, due to the low resolution, the precursor locations often need to be revisited for sufficient sampling, and thus the majority of refixations are immediate returns (lag-2 revisits), when the gaze briefly leaves the precursor location for a saccade aside, but then resumes its inspection (Beck et al., 2006; Godwin et al., 2017).

Neural traces of fragile memory have been found in V4 area of the visual cortex (Sligte et al., 2009). In addition, the distributed patterns of neural activity in the visual cortex mediate the maintenance and precision of representations in short-term memory (Emrich et al., 2013; Hallenbeck et al., 2021; Rademaker et al., 2019). Involvement of short-term memory functions of the visual cortex in refixation behavior is suggested by the predominance of the EEG signature of precursor fixations over the occipital areas (Nikolaev, Ehinger, et al., 2023).

During natural viewing, visual working memory constantly contributes to building dynamic priority maps (Zelinsky & Bisley, 2015). Visual feature templates stored in working memory underlie neural representations of goal states. These goal states construct priority maps by the prioritized weighting of the template features. Working memory is also important for maintaining and monitoring goal states, which supports visual exploration over time. For example, the memory decay at task-relevant precursor locations is likely to be monitored in working memory. When a precursor location in memory decays to a low value, it rises in priority on the priority map, leading to a refixation (Zelinsky et al., 2011). This may result in targets at high salient locations being revisited more frequently (Peterson et al., 2001). Priority maps located in the FEF and IPS (Zelinsky & Bisley, 2015) bias the visual cortex to make it more selective for the relevant visual features according to the task goals (Bressler et al., 2008). In addition, goal-informed saliency maps are topographically represented in the visual cortex (Melloni et al., 2012). Thus, several lines of evidence point to the critical role of memory functions of the visual cortex in refixation behavior. This makes the visual cortex the second candidate structure underlying the maintenance of task-relevant information essential for later refixations.

### High-order refixation guidance by the hippocampus

Refixations are more often executed to task-relevant than to irrelevant locations (Ballard et al., 1995; Wilming et al., 2013; M. Zhang et al., 2022). While FEF and IPS distinguish between targets and distractors during immediate saccade planning (Mirpour et al., 2009), high-order relevance of object and scene locations is derived from general knowledge and understanding of scene context and task goals (Võ & Henderson, 2010), which are provided by interactions between short-term and long-term memory systems (Zelinsky & Bisley, 2015). Here, the hippocampus may play a key role by participating in the online use of short-term memory for saccade guidance informed by long-term memory representations (Kragel & Voss, 2022). In particular, hippocampal theta activity prior to saccade onset predicts whether the target of the next saccade will be selected based on current perception or will be taken from memory and require gaze return (Kragel et al., 2021). In this way, refixations are strategically guided to relevant locations by the hippocampus. Increased sampling of visual information via refixations to relevant locations is critical for information accumulation and robust storage, thus supporting the formation of long-term memory (Kragel et al., 2021; Nikolaev, Bramão, et al., 2023). This suggests that the hippocampal memory system involving the medial temporal lobe (MTL) is the third structure contributing to refixations.

Functional interactions between cortical regions of oculomotor control, visual cortex, and hippocampus are mediated by dense structural connections between these areas (Pierrot-Deseilligny et al., 2004; Shen et al., 2016). Together, these areas may provide a common neural basis for different forms of refixation behavior tailored to specific visual tasks. By returning the gaze to locations with vital information about the visual environment, this brain mechanism makes refixations an essential scaffold for goal-directed visual exploration.

## Outstanding questions and future research

The most intuitive step for future research would be to investigate the neural basis of the three refixation functions identified by eye tracking, which are roughly delineated above. The brain processes underlying these functions have only just begun to unfold, and even the minute aspects of most of them are largely unknown. For example, compensatory refixations are commonly associated with premature interruption of visual processing during fixations leading to inadequate perception. However, the exact causes of this interruption remain unclear. Interruptions may occur for a variety of reasons, including time pressure, the filling up of memory to capacity, distraction that shifts attention to another target, or random forgetting. To understand refixations, it seems advantageous to investigate the neural processes underlying all these possibilities. Nowadays this appears to be feasible by using a smartly designed viewing task combined with EEG–eye-movement coregistration techniques.

Perhaps the most intriguing process that can be illuminated by studying refixation-related neural activity is the construction of mental representations over time during natural viewing. This process involves not only the sampling and encoding of visual information at fixation, with its further accumulation and integration, but also the momentary retrievals of relevant preexisting knowledge and task goals that guide the gaze (Kragel & Voss, 2022). The alternation of encoding and retrieval is supported by rapid exchanges of information between working and long-term episodic memory (Nee & Jonides, 2013; Rose, 2020), when the episodic content is reactivated in working memory (Beukers et al., 2021; Hoskin et al., 2019). In this alternation, refixations correlated with the phase of hippocampal theta activity are thought to provide temporal markers of when short-term memory retrieval guides eye movements (Kragel et al., 2021), as we reviewed above. However, the role of refixations in memory accumulation (Hollingworth & Henderson, 2002; Pertzov et al., 2009; Tatler, Gilchrist, et al., 2005) suggests a much larger contribution of refixations to the construction of memory representations.

The buildup of scene representations across eye movements may involve the integration of multiple local representations that exist in latent, activity-silent neural states (Nikolaev & van Leeuwen, 2019). Unlike traditional working memory, activity-silent representations do not require sustained activity to maintain because they are based on short-term modulation of synaptic weights during encoding (Postle, 2016; Stokes, 2015). Thus, latent representations permit the capacity limits of traditional working memory to be exceeded. This provides the necessary resources to obtain a detailed, fine-grained representation of the whole scene. Reactivated by attentional prioritization, latent representations contribute to the memory buildup according to task demands (van Ede et al., 2017). Here, refixations can be a tool for selective prioritization. Specifically, memory representation at precursor locations may exist in a latent form. Then, refixations can prioritize them by redeploying attention. Since latent representations do not occupy the memory capacity, reactivation by means of refixations would allow achieving a high spatial resolution of the constructed visual scene.

These hypotheses could be tested in a gaze-contingent experiment in which EEG and eye movements are simultaneously recorded in a free-viewing memory paradigm. Latent representations can be decoded from ongoing brain activity with multivariate pattern analysis applied to the brain response evoked by a probe (flash) impulse pinging a hidden neural state (Stokes, 2015; Wolff et al., 2017). During visual exploration of a scene, such probe impulses can be gaze-contingently fired at the precursor locations detected by the online EEG–eye-movement coregistration analysis, which are presumably associated with local latent representations. Comparison of the sequence of latent representations decoded across eye movements could reveal the integration of visual elements during the construction of a memory representation of a scene.

Another field that can be addressed by the refixation-related analysis of neural activity is related to the dynamic nature of refixations. Indeed, refixations are an indispensable part of a highly dynamic scanpath. Therefore, it is essential to investigate refixations with methods that capture the eye-movement dynamics, such as the recurrence quantification analysis described above or MultiMatch, which uses vector-based similarity metrics to compare scanpath similarity (Dewhurst et al., 2012; Johansson et al., 2022). Eye-movement dynamics are supported by the underlying brain dynamics. Therefore, it would be fascinating to study the relationship between the two. We are not aware of any such studies, but attempts to approach them separately have been reported. For example, we have examined the complexity of the brain dynamics underlying fixation-related EEG with methods of the complex systems theory (Seidkhani et al., 2017). In this study, we applied graph theoretical measures to functional connectivity networks derived from EEG time-locked to fixation onset from the visual search dataset described above (Meghanathan et al., 2015, 2019). The local and global topological measures in the alpha frequency band indicated that working memory encoding involves a more segregated mode of operation than retrieval. Future analyses of the nonlinear dynamics of the coupled eye movement and EEG sequences could reveal the evolution of memory states during natural viewing.

An obvious research target that can be approached by analyzing the scanpath dynamics is flexible viewing strategies. For instance, switching between modes is a primary characteristic of multifractality in the distribution of dynamic time series. If oculomotor behavior switches dynamically between exploratory and exploitative modes, eye movements should exhibit multifractal behavior. Indeed, we observed such behavior by applying a multifractal detrended fluctuation analysis to the fixation position series in visual search (Meghanathan et al., 2021). We found weak multifractality, which implies the presence of both small and large fluctuations in the correlation between fixation positions. Large and small fluctuations indicate large and small changes in fixation positions corresponding, respectively, to exploration and exploitation of locations during visual search. Multifractality has been observed not only in the scanpaths of visual search (Vasilyev, 2019) but also in picture comparison and trail making tasks (Freije et al., 2018). It is likely that the exploration and exploitation viewing strategies are associated with distinct modes of neural activity. These strategies, as well as the associated neural activity, may also depend on the holistic and analytic style of the individual’s viewing behavior. Such individual differences need to be considered in future refixation research. Overall, an interesting goal for future research would be to investigate the changes in neural states that lead to different refixation viewing strategies that serve to optimize memory use.

## Conclusions

In reviewing the current scant knowledge about the neural correlates of refixations, we found ourselves at the beginning of a long journey. Refixations occur too frequently to be neglected, or to be treated as a minor topic in vision research. Although the full range of their functions may still be unknown, it is now clear that refixations open a gateway to memory across eye movements. The ongoing memories prioritized by refixations construct a long-term picture of the world. At the same time, preexisting knowledge forces us to look again at the places we have seen. Together, these processes support our goal-directed behavior. It is therefore essential to invest more effort in understanding the neural basis of refixations. We expect research in this area to accelerate as a result of recent advances in methods for the simultaneous analysis of neural activity and eye movements. Indeed, the current state-of-the-art methodological solutions are characterized by high quality and ease of use. This offers vast possibilities for the systematic investigation of various visual tasks involving refixations. As a result, it will finally become clear why we so often look back at what we have already seen. We are certainly looking forward to that!

## Appendix

### Recurrence quantification analysis

Recurrence quantification analysis of the scanpath (Anderson et al. (2013) is the method most directly focused on the examination of refixation behavior. It is based on recurrence plots, which depict fixation sequences on the *x*- and *y*-axes according to fixation rank. To illustrate, in Fig. 3B, the 45 fixations of a trial are represented in both the *x*- and *y*-axes in the order in which they occur, resulting in a symmetric plot. Each point in the plot corresponds to a fixation made on the same location at two different instances of time, therefore, a refixation (Fig. 3B). A point is plotted in when a fixation *f*_*i*_ is recurrent with a fixation *f*_*j*_ *,* determined as

$${r}_{ij}=\left\{\begin{array}{c}1,\kern0.75em d\left({f}_i,\,{f}_j\right)\le \rho, \\ {}0,\kern4em otherwise\end{array}\right.$$

where *d* is the spatial distance between two fixations *f*_*i*_ and *f*_*j*_ and *ρ* is the threshold distance (for example, 2° as discussed above).

In general, the large-scale typology and small-scale texture of the points in a recurrence plot inform us about the dynamics of the system that generated them. For instance, the nonhomogenous texture of points in the recurrence plot in Fig. 3B tells us that the underlying (oculomotor) system is nonstationary (changes with time) and nonrandom. Additional evidence for a nonstationary system arises from the concentration of points in the center of the plot, with relatively sparse points in the top-left and bottom-right corners, indicating the presence of a drift or trend such that recurring states are concentrated in the middle of a trial rather than towards its start or end. These general observations about the recurrence plot, which are likely present consistently across trials of a task, already tell us that (re)fixation behavior is driven by long-term oculomotor dynamics.

When looking at small-scale structures in a recurrence plot, we find finer details of the dynamics of the system, which may help us distinguish between different visual tasks. Isolated points indicate isolated events (refixations) indicating the presence of uncorrelated repeated states (revisited locations), which are rare in the recurrence plot in Fig. 3. In contrast, diagonal lines parallel to the line of symmetry in the plot indicate a trajectory of system states (in this case, fixations) that are similar at different times. Horizontal or vertical lines indicate system states that do not change for some time. Such small-scale temporal patterns visible in the recurrence plot can be quantified with RQA by certain measures, three of which are relevant for refixations and are described below (see Anderson et al., 2013, for details). For all measures, the total number of recurrent points (R) in one half of the symmetric recurrence plot (upper triangle in Fig. 3B) for *N* fixations are calculated as

$$R=\sum_{i-1}^{N-1}\sum_{j=i+1}^N{r}_{ij}.$$

1. Determinism—This is a measure quantifying repeating gaze patterns in the recurrence plot. It is calculated as the proportion of recurrent points that are part of diagonal lines in a recurrence plot:

$$Determinism=100\times \frac{D_L}{R},$$

where, D_L_ is the total number of diagonal lines of length L (2 or more) in the upper triangle of the recurrence plot.

A high value of determinism indicates that more regions were refixated in the same order that they were originally visited, while a low value indicates that refixations were not made in the same order as precursor fixations.

2. Laminarity—This measure is computed as the proportion of dots forming horizontal or vertical lines in a recurrence plot:

$$Laminarity=100\times \frac{H_L+{V}_L}{2R},$$

where H_L_ and V_L_ are, respectively, the total number of horizontal and vertical lines of length L (2 or more) in the upper triangle of the recurrence plot.

Laminarity indicates the frequency of refixations on a single precursor location or vice versa (the frequency of precursor fixations on a single location followed by a single refixation). High laminarity indicates that a location was visited once at one point in time and during a later time period, multiple successive fixations (immediate refixations) were made to the same location resulting in clustered fixations.

3. Center of recurrence mass (CORM)—This measure is computed as the center of gravity of recurrent points from the line of symmetry in a recurrence plot:

$$CORM=100\times \frac{\sum_{i=1}^{N-1}\sum_{j=i+1}^N\left(j-i\right){r}_{ij}}{\left(N-1\right)R}.$$

This measure summarizes how quickly refixations are made after precursor fixations. A high value of CORM implies a large gap or lag between precursor fixations and refixations, whereas a low value indicates that refixations generally occur soon after the precursor fixation.
